# Novel and Promising Therapies for Diabetic Dyslipidemia to Mitigate Residual Cardiovascular Risk

**DOI:** 10.3390/jcm13164915

**Published:** 2024-08-20

**Authors:** Ishwarlal Jialal, Verena Gounden

**Affiliations:** 1 Department of Pathology and Internal Medicine, University of California-Davis, Sacramento, CA 95817, USA; 2 Department of Clinical Biochemistry, University Hospital Galway, H91 YR71 Galway, Ireland; vernenagounden@yahoo.com

Diabetes is a major risk factor for atherosclerotic cardiovascular disease (ASCVD), and the dyslipidemia of diabetes is pivotal in the genesis of this leading cause of increased morbidity and mortality that burdens diabetic patients.

Diabetic dyslipidemia usually manifests as moderate hypertriglyceridemia (HTG), low levels of high-density lipoprotein (HDL) -cholesterol (C), a preponderance of small dense low-density lipoprotein (LDL) particles, and elevated levels of apolipoprotein B (apoB) [1,2,3]. Both the AHA/ACC and ESC guidelines have made LDL cholesterol the primary target of therapy, with the statin class of drugs being the recommended cornerstone of pharmacotherapy in addition to therapeutic lifestyle changes [4,5]. The available therapies endorsed by both guidelines include bile acid sequestrants, statins, proprotein convertase subtilisin/kexin9 (PCSK9) inhibitor therapies, and ezetimibe in combination with statin therapy [1,2,3,4,5]. In addition to these monoclonal antibodies against PCSK9, Inclisiran, a small interfering RNA (siRNA) is very efficacious in lowering LDL cholesterol and is well tolerated with twice yearly injections [2,3]. However, clinical trials showing a reduction in ASCVD events with Inclisiran are still ongoing. In patients with ASCVD with well-controlled LDL-C levels on statin therapy and TG levels between 135 and 499 mg/dL, icosapent ethyl (4.0 g/d) alone was shown in a single study to reduce ASCVD events and thus could be an additional therapy [1,2,3]. In this editorial, we discuss the roles of first-in-class ATP-citrate lyase inhibitor, bempedoic acid and promising therapeutics directed against apolipoprotein C-III, Angiopoietin-like 3 (ANGPTL3) and lipoprotein (a) as part of the therapeutic regime of the future.

Bempedoic acid (BA) is an inhibitor of ATP-citrate lyase (ACL), upstream of HMG-CoA reductase in the cholesterol biosynthetic pathway, resulting in a decrease in LDL-cholesterol and apolipoprotein B [6]. It is a prodrug that is activated in the liver to bempedoyl-CoA, the active metabolite that inhibits ACL, resulting in a decrease in cholesterol biosynthesis, which leads to an increase in liver LDL receptors and a lowering of plasma LDL-C.

The CLEAR Outcomes trial was a large trial of 13,970 patients who were unable or unwilling to take statin therapy due to unacceptable adverse reactions (statin intolerance) and had ASCVD or were at high risk for ASCVD [7]. An equivalent number of patients were assigned to BA (180 mg/day orally) or placebo. The patients were followed up for a median duration of 3.4 years. The primary endpoint was the composite of major adverse ASCVD events: death from cardiovascular causes, non-fatal myocardial infarction, non-fatal stroke, and coronary revascularizations. Only 23% of patients were on statin therapy. Germane to this editorial, patients with diabetes comprised 45% of the study cohort. At 6 months, the mean LDL-C was 107 mg/dl in the BA group compared to 136 mg/dL in the placebo group, a significant 21.7% reduction. Over the duration of the trial, the time-average LDL-C reduction with BA was 15.9%. The percent reduction on hsCRP was 21.6% at 6 months, and the lower hsCRP levels in the BA were persistent over the duration of the trial.

There was a significant reduction in the composite primary end point with a hazards ratio (HR) of 0.87 and 95% confidence intervals (CI) of 0.79 to 0.96, *p* = 0.004. As previously reported, hyperuricemia, gout, increased creatinine levels, and cholelithiasis were more common in the BA group. 

In a subsequent report, the authors examined pre-specified end points, which included the effect of BA on ASCVD events, new-onset diabetes, and glycated hemoglobin levels [8]. They confirmed in the diabetic sub-cohort (*n* = 6373) that BA reduced LDL-C and hsCRP levels. Most importantly, it reduced ASCVD events by 17 percent, with no increase in new-onset diabetes or worsening in glycated hemoglobin levels.

Statin therapy is still the gold standard for LDL-C lowering therapy. However, BA may have a unique role in patients who cannot tolerate or are unwilling to take statins. Another advantage of BA over statins is the prevention of new onset diabetes or the worsening of hyperglycemia. Additionally, BA, in combination with ezetimibe, is an attractive option for patients who have declined treatment with injectable non-statin therapies [6].

Hypertriglyceridemia is a recognized independent risk factor for cardiovascular disease [9]. ApoC–III is a 79 amino acid glycoprotein encoded by the *APOC3* gene. It is predominantly synthesized in the liver and enterocytes and is present in most lipoproteins in different proportions. ApoC-III is an inhibitor of lipoprotein lipase (LPL) and thus plays a significant role in the metabolism of triglyceride-rich lipoproteins (TRLs). The action of apoC-III on LPL decreases the uptake of TRLs by the LPL pathway. Actions of apoC-III affecting triglycerides include inhibition of LPL-mediated lipolysis, promoting assembly and secretion of VLDL, and affecting Apo-E-mediated uptake of VLDL by the liver. The major effect of apoC-III actions results in increased plasma triglycerides and TRLs [2,3,10].

Various studies have demonstrated a link between apoC-III levels and increased risk for ASCVD, independent of its effect on triglyceride levels. Furthermore, loss of function mutations in apoC-III are associated with lower TG levels and decreased risk for ASCVD [2,3]. As such, it has been identified as a pharmacological target for therapy to reduce TGs and ASCVD risk. This includes the development of antisense oligonucleotides (ASO) which are single-stranded oligonucleotides that regulate the expression of the targeted protein, in this case apoC-III, by binding to relevant areas of the messenger RNA (mRNA). Volanesorsen, an ASO, was demonstrated to decrease plasma apoC-III levels by over 80% and triglycerides by 69% in a randomized placebo-controlled trials of patients with multifactorial chylomicronemia syndrome including type 2 diabetics. Insulin sensitivity was also noted to be increased in the volanesorsen-treated group, and this correlated with the decrease in apoC-III and TG levels. The European Medicines Agency (EMA) has given conditional authorization for the use of volanesorsen for the treatment of familial chylomicronemia syndrome based on positive results from the phase 3 studies. Thrombocytopenia has been reported in a significant number of trial patients in the Volanesorsen group, and it also raises LDL-C levels. Additionally, evidence is limited for its utility in a broader patient population and favorable impact on cardiovascular outcomes [3,11,12]. Olezarsen is a hepatic-specific modified ASO via conjugation to N-acetyl-galactosamine which targets the asialoglycoprotein receptor on hepatocytes and thus binds more specifically to apoC-III [2,3]. It has been reported to have an improved safety profile and tolerability over volaneosorsen. Both volanesorsen and olezarsen are administered via subcutaneous injections; however, olezarsen requires lower doses and less frequent dosing to achieve similar reductions in TG levels and increases in HDL-C. A recent phase 2 trial demonstrated a reduction of apoB, apoC3, and non-HDL-C with no significant change in LDL-C [13]. Outcome data from several phase 3 studies of olezarsen are eagerly awaited [3,13].

Plozasiran is an N-acetylgalactosamine-conjugated small interfering RNA (siRNA) that acts by decreasing the expression of apoC-III in the liver. Findings of a phase 1 study have demonstrated substantial dose-dependent reductions in both apoC-III and TG. The results of phase 2 clinical trials with promising findings have been recently published [14]. 

Angiopoietin-like 3 (ANGPLT3) is a 70-kDa glycoprotein secreted by the liver [3]. ANGPLT3 was revealed to have a role in lipoprotein metabolism by the identification of deletions and loss of function variants in *ANGPLT3*, which were associated with lower levels of plasma TGs and LDL-C and a noted reduction in ASCVD risk [3,15]. ANGPLT3 acts to inhibit LPL and endothelial lipase (EL) in various tissues, increasing TGs and TRLs in circulation. Novel therapeutic agents to inhibit ANGPLT3 have been developed. Evinacumab is a human monoclonal antibody developed using genetically engineered mice with a humanized immune system. When administered subcutaneously or intravenously, evinacumab binds to ANGPLT3, thus inhibiting its effect on LPL and EL activity. This results in a decrease in plasma TGs, as well as a decrease in both LDL-C and apoB independent of the classical LDL receptor-mediated pathway [3,15]. 

In 2021, Evinacumab was approved by the EMA and FDA for use in homozygous familial hypercholesterolemia. In contrast to other newer treatments for hypercholesterolemia (PCSK9 inhibitors), Evinacumab has been reported to have greater efficacy in lowering plasma TGs and TRLs [3]. However, long-duration cardiovascular outcome trials are required to assess the impact on CVD and safety.

Trials for ASO (Vupanorsen), developed to inhibit ANGPLT3 synthesis, were discontinued in 2022 due to the limited beneficial effect on TG and non-HDL-C levels. Additionally, dose-dependent increases in liver fat deposition and elevations in liver enzymes were observed [3]. ARO-ANGPTL3 is a siRNA developed against ANGPTL3 mRNA to reduce expression by hepatocytes. The ARCHES-2 trial investigators recently published their phase 2 findings for ARO- ANGPTL3,Zodasiran, which demonstrated at the highest dose (200 mg) compared to placebo a 63% and 22% reduction in TGs and apoB, respectively, after 24 weeks of treatment [16]. Also there was a 20% reduction in LDL-C and 36% decrease in non-HDL-C. Solbinsiran is another siRNA that has been developed that targets ANGPLT3 expression by hepatocytes. 

Other anigiopoietin-like proteins namely, ANGPLT-4 and ANGPLT-8, have also been described to have influence on LPL activity either alone or together with ANGLPT-3. In particular, in animal studies the blocking of ANGPLT8 by antibodies resulted in decreased serum TGs and increased HDL-C, making this another possible future candidate therapeutic target [17]. Vaccines targeting the LPL-binding domain of ANGPLTs as well as gene editing therapy via CRISPR/Cas 9 are in preclinical studies [17].

An elevated level of lipoprotein(a) is a risk factor for ASCVD based on epidemiological, genome wide association and Mendelian randomization studies [18,19]. Exciting therapies are now available to target the LPA gene via ASO and siRNA technologies that result in a substantial reduction in Lp(a) levels [20,21]. Some of these therapies are already in phase 3 trials. If they yield positive results on ASCVD events with minimal side effects, they could emerge as adjunctive therapies for diabetics also.

In conclusion, since ASCVD is the major cause of mortality in diabetes, it is imperative to continue to target this major problem. Thus, in addition to a healthy lifestyle, smoking cessation, good glycemic control, adequate management of blood pressure, and lowering LDL-C, additional therapies are required. To this end, bempedoic acid is an attractive addition for patients who are statin-intolerant to lower both LDL-C and hsCRP levels. In addition, since most patients with type 2 diabetes are at high or very high risk, requiring at least a 50% reduction in LDL-C, with target goals of <70 and <55 mg/dL, respectively, combination therapy with ezetimibe, PCSK9 inhibitors, and bempedoic acid appears to be a prudent first line if high-dose statin cannot be tolerated. For severe HTG, to avert the risk of pancreatitis, in addition to fibrates and omega-3 fatty acids, apoC-III inhibitors, such as Olezarsen, could emerge as an excellent choice if the clinical trials reveal sufficient safety and efficacy, with the added hope of reducing ASCVD events. However, the more compelling choice if the ongoing clinical trials show a reduction in ASCVD events will be ANGPTL-3 inhibitors. It is premature to recommend this latter two class of drugs without demonstrating both a reduction in ASCVD events and safety in large clinical trials.

## Data Availability

The data are available from the senior author for review upon reasonable request.
